# Correlation of Antimuscarinic Acetylcholine Receptor Antibody Titers With Pemphigus Disease Activity at Baseline and Following Phase I Pulse Therapy

**DOI:** 10.7759/cureus.85679

**Published:** 2025-06-10

**Authors:** Biswanath Behera, Aparna Palit, Suchanda Sahu, Soumya S Sahoo, Madhusmita Sethy

**Affiliations:** 1 Department of Dermatology, Venereology and Leprology, All India Institute of Medical Sciences, Bhubaneswar, Bhubaneswar, IND; 2 Department of Dermatology and Venereology, All India Institute of Medical Sciences, Kalyani, Kalyani, IND; 3 Department of Biochemistry, All India Institute of Medical Sciences, Bhubaneswar, Bhubaneswar, IND; 4 Department of Community and Family Medicine, All India Institute of Medical Sciences, Bathinda, Bathinda, IND; 5 Department of Pathology, All India Institute of Medical Sciences, Bhubaneswar, Bhubaneswar, IND

**Keywords:** antimuscarinic acetylcholine receptor antibody, dexamethasone-cyclophosphamide/azathioprine, disease activity, pemphigus, phase i therapy

## Abstract

Background

Pemphigus is a rare autoimmune blistering disorder characterized by involvement of the skin and mucous membranes, primarily due to autoantibodies targeting desmogleins. Emerging evidence has pointed to a potential pathogenic role of antimuscarinic acetylcholine receptor (anti-M-AChR) antibodies and reported a correlation between their titers and pemphigus disease activity. However, data on this association, particularly in relation to different phases of pulse therapy in the Indian context, remain limited.

Aim

This study aimed to evaluate the correlation between anti-M-AChR antibody titers and pemphigus disease activity at baseline and after Phase I of dexamethasone-cyclophosphamide/azathioprine pulse therapy.

Materials and methods

This prospective longitudinal observational study included newly diagnosed cases of pemphigus confirmed through histopathology and direct immunofluorescence from April 2019 to March 2021. Pemphigus Disease Area Index (PDAI) scores were recorded, and eligible patients received dexamethasone-cyclophosphamide or dexamethasone-azathioprine pulse therapy as appropriate.

Results

A total of 29 patients were enrolled: 23 (79%) with pemphigus vulgaris, five (17%) with pemphigus foliaceus, and one (3.4%) with pemphigus erythematosus. Patient ages ranged from 21 to 69 years, with a male-to-female ratio of 0.7:1. By the end of Phase I therapy, 20 patients (69%) completed follow-up. The mean baseline anti-M-AChR antibody titer was 76.48 ± 48.12 U/mL, which decreased to 53.61 ± 30.97 U/mL after Phase I. At that point, PDAI scores showed a moderate correlation with anti-M-AChR antibody levels (r = 0.51, p = 0.02). The reduction in antibody titers from baseline to the end of Phase I was statistically significant (p = 0.05).

Conclusions

While serum anti-M-AChR antibody titers may not reliably indicate disease activity at the time of diagnosis, their levels appear to reflect changes in disease activity following treatment. This suggests their potential use as a prognostic marker during the course of therapy.

## Introduction

Pemphigus is a rare group of autoimmune blistering disorders affecting the skin and mucous membranes, characterized by the formation of antibodies against cell surface adhesion molecules - specifically desmoglein 1 (Dsg1) and desmoglein 3 (Dsg3). The disease is broadly classified into two major variants: pemphigus vulgaris (including the vegetans subtype) and pemphigus foliaceus (including the erythematosus subtype). The primary autoantigens in both forms are Dsg1 and Dsg3, which are considered the most important pathogenic targets in pemphigus. Circulating intercellular antibody titers often parallel disease activity. Among these, anti-Dsg1 antibody titers tend to show a stronger correlation with disease course than anti-Dsg3 antibody titers in patients with pemphigus [1-3].

Recent studies have proposed a multiple-hit hypothesis, suggesting that acantholysis in pemphigus is mediated not only by desmoglein but also by non-desmoglein antigens. Among these, antimuscarinic acetylcholine receptor (anti-M-AChR) antibodies have been shown to play a significant role in pemphigus pathogenesis [4]. Acetylcholine (ACh) plays an important role in keratinocyte cell-cell and cell-matrix adhesion in the stratified squamous epithelium through ACh receptors (AChRs). Autoantibodies targeting epidermal and/or mucosal keratinocyte AChRs have been identified in patients with pemphigus [5].

Anti-M-AChR antibodies are believed to contribute to the cutaneous activity in pemphigus vulgaris by interfering with the physiological regulation of cadherin expression and function mediated by AChRs. This interference results in impaired intercellular adhesion between keratinocytes, causing cell detachment and acantholysis [4]. Supporting this, in vitro studies have demonstrated that cholinomimetic drugs can reverse antibody-mediated acantholysis, and a patient with both pemphigus vulgaris and myasthenia gravis was successfully managed with pyridostigmine bromide for five years [6]. Furthermore, the role of anti-AChR antibodies in pemphigus pathogenesis is supported by observations of intraepidermal splits and acantholysis in pemphigus vulgaris patients lacking anti-Dsg1/3 antibodies, as well as by disruptions in normal keratinocyte morphology following prolonged exposure to anti-AChR autoantibodies [6,7]. Several studies have also reported a correlation between anti-M-AChR antibody titers and pemphigus disease activity [8,9].

In contrast to treatment protocols in developed countries, dexamethasone-cyclophosphamide pulse (DCP) therapy remains the cornerstone of pemphigus management in India. For younger patients or those of reproductive age, azathioprine is often substituted for cyclophosphamide (dexamethasone-azathioprine pulse (DAP) regimen). Pulse therapy consists of four defined phases. Phase I involves administration of DCP, daily oral cyclophosphamide (50 mg), and a tapering dose of oral steroids until the patient is clinically lesion-free and no longer dependent on steroids. Phase II includes nine months of DCP with daily cyclophosphamide. Phase III consists of cyclophosphamide alone (50 mg daily), and Phase IV is the post-therapy follow-up period. DCP or DAP pulses are administered at fixed 28-day intervals [10-13].

While a previous Indian study explored the correlation between anti-M-AChR antibody titers and pemphigus disease activity, it did not specifically address the variations in these titers during different phases of pulse therapy [9]. This study aims to address that gap by investigating whether anti-M-AChR antibody titers correlate with disease activity both at diagnosis and after Phase I therapy and whether these titers can serve as a prognostic marker for treatment response.

The objective of this study was to evaluate the correlation between anti-M-AChR antibody titers and disease activity in pemphigus patients at the time of diagnosis and following completion of Phase I dexamethasone-cyclophosphamide/azathioprine pulse therapy and to assess whether changes in these titers may serve as an indicator of therapeutic response.

## Materials and methods

This prospective longitudinal observational study was conducted after obtaining approval from the institute’s ethics committee (T/IM-F/18-19/19). The study included all newly diagnosed cases of pemphigus presenting with active mucosal and/or cutaneous lesions, confirmed through histopathology and direct immunofluorescence (DIF), between April 2019 and March 2021. Patients who had previously received DCP/DAP therapy or rituximab, as well as pregnant or lactating patients in whom DCP/DAP therapy was contraindicated, were excluded.

The objective of the study was to correlate anti-M-AChR antibody titers with pemphigus disease activity at baseline and after completion of Phase I dexamethasone-cyclophosphamide/azathioprine pulse therapy and to evaluate its potential role as a prognostic marker in the treatment of pemphigus. Patients who met the inclusion and exclusion criteria were recruited by AP from the dermatology outpatient and inpatient departments after obtaining informed consent.

Demographic and clinical data - including age, sex, age of onset, disease duration, treatment history, and comorbidities - were recorded using a standardized pro forma. Disease activity was assessed using the Pemphigus Disease Area Index (PDAI), scored by BB, who was blinded to the antibody titer results [14]. Two skin punch biopsies were taken from each patient: one for histopathology and another for DIF, both analyzed by MS. Eligible patients received either DCP pulse therapy (intravenous dexamethasone 100 mg in 500 mL of 5% dextrose for three consecutive days, intravenous cyclophosphamide 500 mg on the second day, and oral cyclophosphamide 50 mg daily) or, in the case of patients of reproductive age, DAP therapy (oral azathioprine 50 mg twice daily instead of cyclophosphamide). Pulse therapy was repeated every 28 days, and Phase I was considered complete when cutaneous and mucosal lesions had fully healed without any breakthrough lesions between treatment cycles.

**Figure 1:**
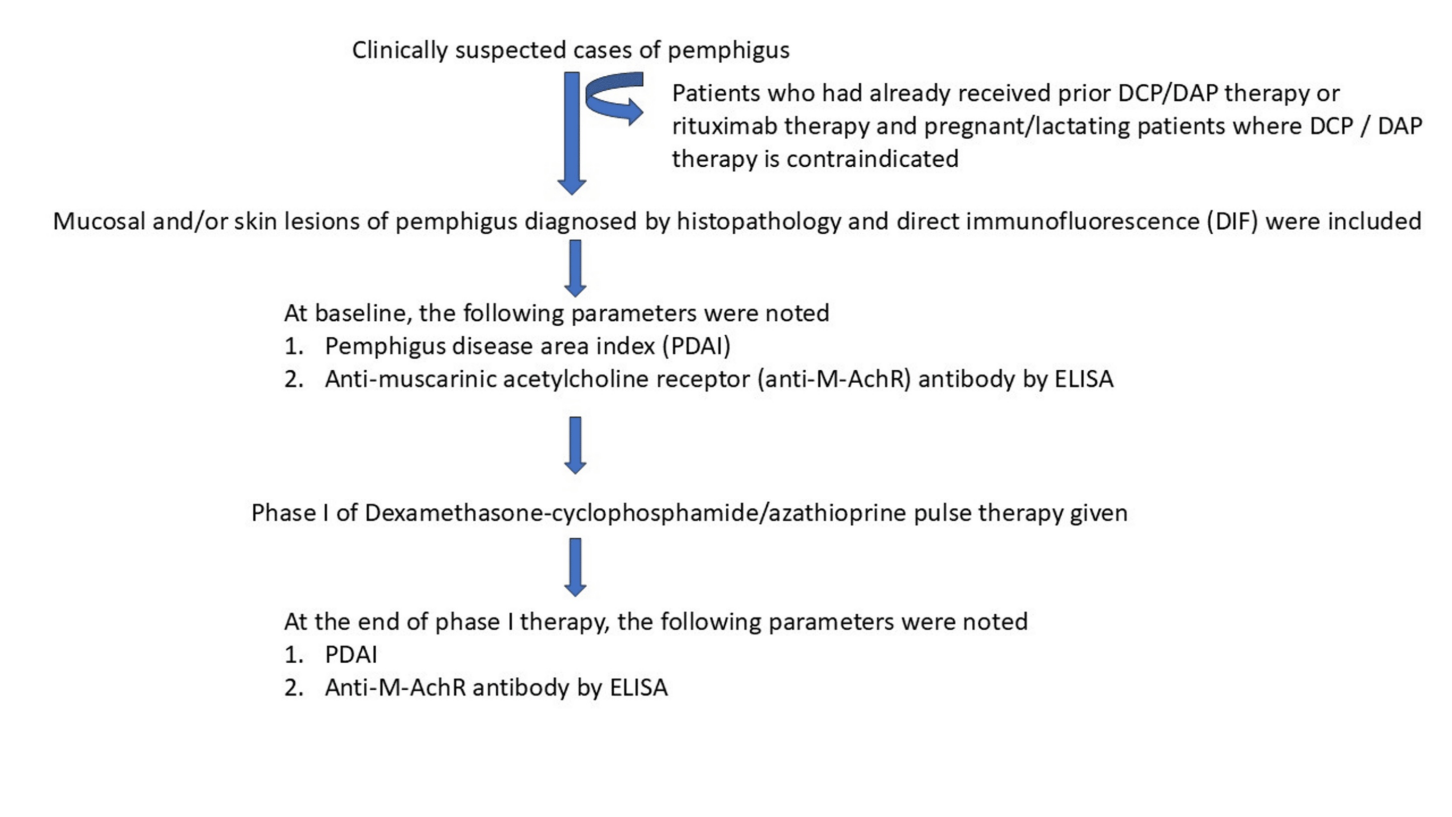
Brief study procedure Anti-M-AChR, antimuscarinic acetylcholine receptor; DAP, dexamethasone-azathioprine pulse; DCP, dexamethasone-cyclophosphamide pulse; DIF, direct immunofluorescence; ELISA, enzyme-linked immunosorbent assay; PDAI, Pemphigus Disease Area Index

Venous blood samples (5 mL) were collected from each patient at baseline and at the end of Phase I. The samples were allowed to clot at room temperature and then centrifuged at 2,000 rpm for 15 minutes. The resulting serum supernatant was stored at -80°C. Anti-AChR antibody titers were measured at both time points using the enzyme-linked immunosorbent assay (ELISA) method, conducted by SS, with a commercial ELISA kit specific for human muscarinic acetylcholine receptor autoantibodies (Cusabio, Houston, TX, USA). An outline of the study procedure is illustrated in Figure 1.

Statistical analysis

Anticipating a moderate correlation due to the exploratory nature of the study, we calculated a required sample size of 29 participants to detect a correlation coefficient of 0.5 between PDAI scores and anti-M-AChR antibody titers. This calculation was based on a two-tailed alpha level of 0.05 and a power of 80% (β = 0.20), using the following formula:

\[ n = \left( \frac{Z_{1 - \alpha/2} + Z_{1 - \beta}}{0.5 \cdot \ln\left(\frac{1 + r}{1 - r}\right)} \right)^2 + 3 \]

Substituting the values,

\[ n = \left( \frac{2.8}{0.5 \cdot \ln\left(\frac{1 + 0.5}{1 - 0.5}\right)} \right)^2 + 3 \\ = \left( \frac{2.8}{0.5 \cdot 1.0986} \right)^2 + 3 \\ = \left( \frac{2.8}{0.5493} \right)^2 + 3 \\ = 5.096^2 + 3 \\ \approx 25.97 + 3 = 28.97 \]

Categorical data were presented as frequencies and percentages. For continuous variables, normality was tested and the results were expressed either as means with standard deviation or medians with range, as appropriate. Spearman rank correlation was employed to assess the relationship between PDAI and anti-M-AChR antibody titers at baseline and after Phase I. Changes in titers were evaluated using the Wilcoxon signed-rank test. A p-value less than 0.05 was considered statistically significant.

## Results

A total of 29 patients who met the inclusion and exclusion criteria were enrolled in the study. The patients ranged in age from 21 to 69 years, with 17 women and 12 men (male-to-female ratio: 0.7:1). Among the 29 cases, 23 (79%) were diagnosed with pemphigus vulgaris, five (17%) with pemphigus foliaceous, and one (3.4%) with pemphigus erythematosus. The duration of disease at presentation ranged from two to 14 months. Oral mucosal involvement was observed in 26 patients (89%), while genital mucosal involvement was noted in six patients (21%).

At the end of Phase I, follow-up data were available for 20 patients (69%), while the remaining nine were lost to follow-up due to the COVID-19 pandemic (Table 1). Of the nine lost to follow-up, six were female and three were male; seven had pemphigus vulgaris and two had pemphigus foliaceous. No patients were lost to follow-up due to non-compliance with therapy.

**Table 1:** Demographic and clinical features of pemphigus cases

Sl. no.	Features	Total cases (N = 29; 100%)
1	Age (years)	Range: 21-69
2	Gender	
	Male	12 (41%)
	Female	17 (59%)
3	Final diagnosis	
	Pemphigus vulgaris	23 (79%)
	Pemphigus foliaceous	5 (17%)
	Pemphigus erythematosus	1 (3.4%)
4	Duration of disease	Range: 2-14 months
5	Site of lesions	
	Oral mucosa	26 (89%)
	Genital mucosa	6 (21%)

The mean PDAI score at baseline was 28.59 ± 7.12, which decreased to 4.16 ± 7.55 at the end of Phase I. The average anti-M-AchR antibody titer was 76.48 ± 48.12 U/mL at baseline and 53.61 ± 30.97 U/mL following Phase I of pulse therapy.

**Figure 2:**
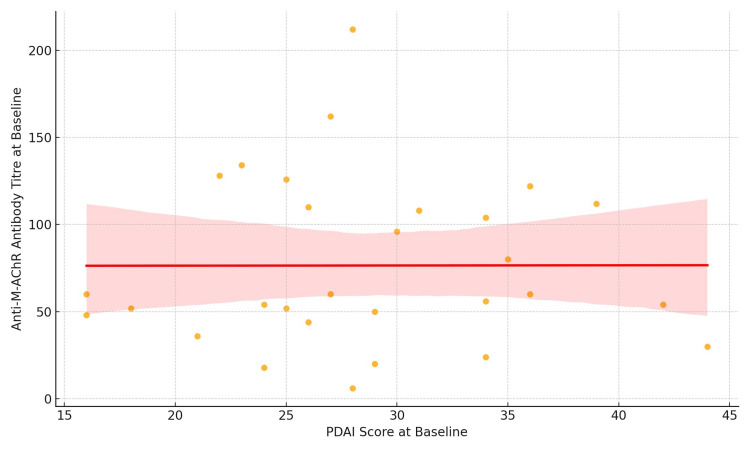
Scatter plot showing the correlation between PDAI and anti-M-AchR antibody titers at baseline Anti-M-AChR, antimuscarinic acetylcholine receptor; PDAI, Pemphigus Disease Area Index

At baseline, there was a weak correlation between PDAI scores and anti-M-AchR antibody titers (r = 0.045), which was not statistically significant (p = 0.81) (Figure 2).

**Figure 3:**
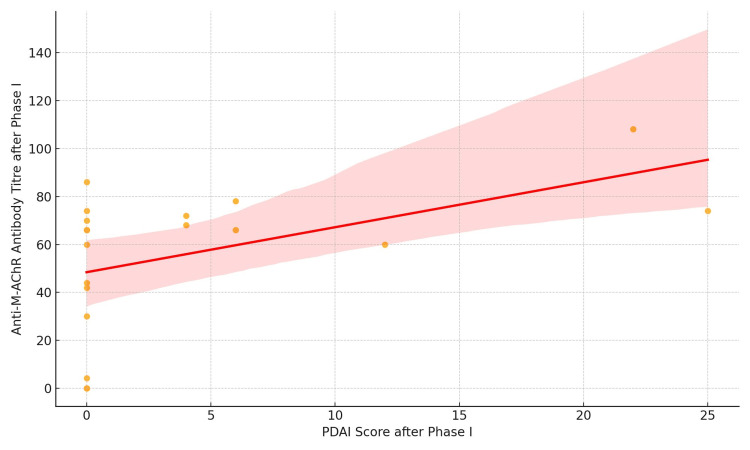
Scatter plot showing the correlation between PDAI and anti-M-AChR antibody titers following Phase I pulse therapy Anti-M-AChR, antimuscarinic acetylcholine receptor; PDAI, Pemphigus Disease Area Index

By the end of Phase I, a moderate correlation was observed between PDAI scores and anti-M-AchR antibody titers (r = 0.51, p = 0.02), as shown in Table 2 and Figure 3.

**Table 2:** Correlation between PDAI and anti-M-AChR antibody titers ^*^ Statistically significant Anti-M-AChR, antimuscarinic acetylcholine receptor; PDAI, Pemphigus Disease Area Index

Time point	Correlation coefficient, r (p-value)
PDAI at baseline	0.045 (0.815)
PDAI after Phase I pulse therapy	0.511 (0.025)^*^

**Figure 4:**
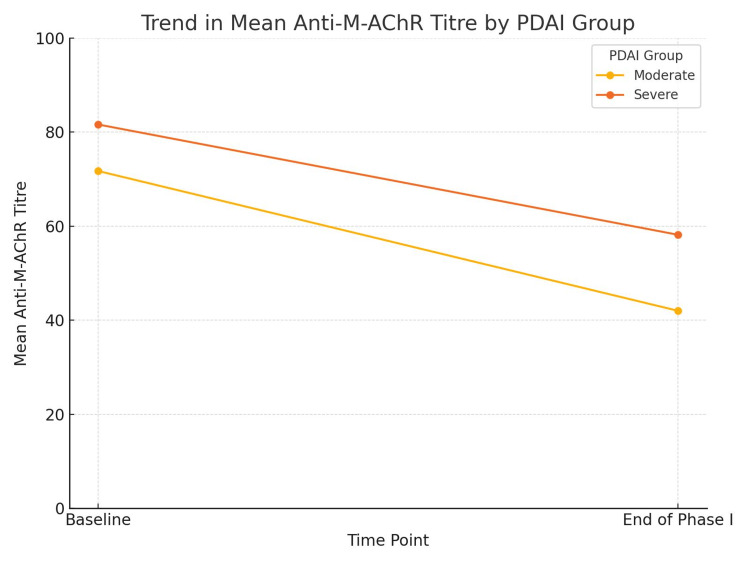
Trend in mean anti-M-AChR antibody titers by PDAI group following Phase I pulse therapy Anti-M-AChR, antimuscarinic acetylcholine receptor; PDAI, Pemphigus Disease Area Index

The change in anti-M-AChR antibody titers from baseline to the end of Phase I pulse therapy was analyzed in 20 patients using the Wilcoxon signed-rank test. A statistically significant reduction in anti-M-AChR antibody titers was observed at the end of Phase I compared to baseline (p = 0.05), as shown in Table 3 and Figure 4, based on pairwise analysis of the 20 matched samples.

**Table 3:** Changes in PDAI and anti-M-AChR antibody titers at the end of Phase I pulse therapy ^*^ Statistically significant Anti-M-AChR, antimuscarinic acetylcholine receptor; PDAI, Pemphigus Disease Area Index

Parameter	Baseline (n = 29)	End of Phase I of pulse therapy (n = 20)	p-value
PDAI	28.59 ± 7.12	4.16 ± 7.55	<0.001^*^
Anti-M AchR antibody titer (U/ml)	76.48 ± 48.12	53.61 ± 30.97	0.05^*^

## Discussion

Pemphigus is a rare, life-threatening autoimmune vesicobullous disease that remains a therapeutic challenge. While diagnosis is generally straightforward, the key difficulty lies in determining the most appropriate type and duration of therapy. Among the various available regimens, DCP/DAP therapy is the preferred treatment in India and comprises four well-defined phases. Despite adherence to these protocols, a substantial number of patients relapse after the apparent successful completion of therapy [10,11]. This issue is compounded by the lack of an objective method to predict relapse or treatment failure with DCP/DAP therapy. Although some studies have shown that anti-desmoglein (anti-Dsg) antibodies correlate with disease activity and may serve as predictors of relapse - specifically, anti-Dsg1 correlating with the severity of cutaneous lesions and anti-Dsg3 with mucosal involvement [3,15-19] - these studies have not tracked antibody titers through each phase of pulse therapy.

In our study, we found only a weak correlation between baseline anti-M-AChR antibody titers and disease activity, as measured by the PDAI. Several factors may account for this weak correlation: variability in anti-M-AChR antibody levels between patients; the possibility that this antibody is not the sole or primary pathogenic driver of disease; and the limitation of single-point measurement, which may not capture peak immunologic activity. Furthermore, PDAI reflects clinical manifestations such as blisters, erosions, and new erythema, which may be influenced not only by antibody-mediated pathology but also by non-antibody mechanisms, the patient’s healing capacity, and secondary infections.

However, at the end of Phase I, a moderate and statistically significant correlation was observed between PDAI and anti-M-AChR antibody titers. Additionally, there was a significant decline in both PDAI and antibody titers following Phase I therapy. Although current data are insufficient to definitively conclude that PDAI reduction is directly caused by decreased anti-M-AChR production, the parallel decline in both variables in our study suggests that PDAI may independently reflect anti-M-AChR activity.

Previous studies have reported the presence of anti-M-AChR antibodies in approximately 85% of pemphigus cases, with levels correlating significantly with disease severity at diagnosis and during follow-up. However, with the exception of one study, there is limited comparative data on anti-M-AChR and anti-Dsg antibodies in pemphigus [9]. While anti-Dsg1 and anti-Dsg3 antibodies are well-established pathogenic, diagnostic, and prognostic markers, the clinical relevance of anti-M-AChR antibodies remains less clear. The diagnostic utility of anti-M-AChR titers has not been established, and it is uncertain whether elevated levels are pathogenic or represent an epiphenomenon [8,20,21]. A practical consideration is that testing for both anti-Dsg1 and anti-Dsg3 can be costly, whereas anti-M-AChR antibody titers might serve as a single, more affordable marker of disease activity. In our study, anti-M-AChR antibodies were detectable in all patients at baseline.

A previous study from India demonstrated a positive correlation between anti-M-AChR titers and disease activity at baseline and post-treatment [9]. In contrast, our findings showed a significant correlation only after Phase I of pulse therapy. That study reported that titers above 19.53 ng/dL were associated with disease presence, with 100% sensitivity and specificity. They also reported antibody levels much higher than those found by Tirado-Sánchez et al. [8]. In our cohort, titers were lower than those reported by Lakshmi et al. but higher than those in the study by Tirado-Sánchez et al. [8,9]. Lakshmi et al. also reported a positive correlation between anti-M-AChR and anti-Dsg1 antibody titers, though the relationship was not statistically significant [9]. Due to the lack of a control group, we could not calculate the sensitivity and specificity of anti-M-AChR titers in our study.

Our findings support the potential role of anti-M-AChR antibody as a marker of treatment response, as indicated by the significant reduction in titers following Phase I therapy. While immunosuppressive therapy is expected to reduce antibody levels, the concurrent and statistically significant decline in PDAI suggests that anti-M-AChR titers may serve as a prognostic, although not diagnostic, marker. Monitoring these titers serially could offer insight into changes in disease activity.

Limitations

This study has several limitations, including a relatively small sample size, recruitment from a single geographic region, and the absence of both a control group and comparative anti-Dsg antibody titers. Additionally, nine participants were lost to follow-up at the end of Phase I due to the COVID-19 pandemic, which could increase the risk of a type II error.

## Conclusions

This study investigates the role of serum anti-M-AChR antibody titers in pemphigus. We conclude that while these titers may not reflect disease activity at the time of diagnosis, they may serve as prognostic markers to monitor changes in disease activity following treatment. However, larger studies with more diverse populations and comparisons with anti-Dsg titers are needed to validate our findings and further explore the pathogenic and diagnostic potential of anti-M-AChR antibodies in pemphigus.
